# MicroRNA‐345‐5p regulates depression by targeting suppressor of cytokine signaling 1

**DOI:** 10.1002/brb3.1653

**Published:** 2020-07-30

**Authors:** Yulan Liu, Jun Yu, Xinrui Wang, Jicheng Dong

**Affiliations:** ^1^ Psychiatric Department Qingdao Mental Health Center, Qingdao university Qingdao City China

**Keywords:** apoptosis, CDK6, depression, miR‐345‐5p, SOCS1

## Abstract

**Background/Aims:**

MicroRNA(miR)‐345‐5p plays a key role in various cellular functions. However, the function of miR‐345‐5p in resistant depression (TRD) is unclear. The aim of this study was to evaluate the role and mechanism of miR‐345‐5p in the treatment of resistance depression (TRD).

**Methods:**

RT‐qPCR was used to detect the expression of miR‐345‐5p in BV‐2 microglia. CCK‐8 method and flow cytometry were used for cell viability and apoptosis of microglia. Target gene prediction and screening, and luciferase reporter assays were used to verify the downstream target gene of miR‐345‐5p. Western blot was used to analyze the protein expression of related proteins.

**Results:**

miR‐345‐5p increased the cell viability of BV‐2 microglia and the expression level of pro‐inflammatory cytokines. In addition, the conditioned medium of microglia treated with miR‐345‐5p reduced the cell viability of HT22 hippocampal cells and caused S‐phase arrest. The miR‐345‐5p‐treated microglia induced apoptosis by regulating the expression levels of Bax, Bcl‐2, pro‐caspase‐3, and cleaved caspase‐3. Furthermore, SOCS1 was a direct target of miR‐345‐5p, and overexpression of SOCS1 was able to reverse the proapoptotic effect of miR‐345‐5p on activation of microglia on hippocampal neurons.

**Conclusion:**

miR‐345‐5p induced inflammatory damage in hippocampal neurons by activating microglia. MiR‐345‐5p may be an effective target for TRD therapy.

## Graphical abstract

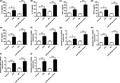

## 1 BACKGROUND

Resistant depression (TED) is the most common mental illness (Sowa‐Kućma et al., 2018). About 350 million patients have become a serious public health problem all over the world (Cusin et al., 2017). Studies have shown that TED is also associated with other diseases, such as osteoporosis, diabetes, and cerebral ischemia (Grønli & Wynn, 2013; Nierenberg, Mcintyre, & Sachs, 2015). Even so, the researchers’ understanding of depression is still very limited. In recent years, people have found the structural changes and functional impairment in some brain regions of patients with depression based on the brain imaging and autopsy reports of depression patients (Buckner et al., 2018; Halappa et al., 2018). As the research progressed, neuroplastic changes have provided new evidence for explaining the delay in antidepressant effects (Koskimäki, Matsui, Umemori, Rantamäki, & Castrén, 2015). It is believed that the function of neurons plays a dominant role in neuroplasticity, while the function of other cells is neglected (Wu, Muthuchamy, & Reddy, 2017). There is increasing evidence that many immunocompetent cells in the central nervous system, especially microglia, play a big role the development of depression by affecting neural plasticity (Ivanova et al., 2007; Singhal & Baune, 2017).

Studies have shown that there are multiple levels of apparent regulation of depressive disorders (Antipova, Krasnov, & Trofimova, 2015; Sabunciyan et al., 2012). Among them, noncoding RNA regulation is the most important regulatory pathway (Zhu, Li, Li, Zhang, & Wang, 2019). miRNA is widely involved in pivotal biological processes, for example, physiological function regulation through cleavage degradation or translational inhibition of the target gene mRNA (Ammt, Cho, Choi, Hong, & Kim, 2018). A miRNA can act on hundreds of target genes, and different miRNAs can also be combined with the same m RNA target gene (Fang et al., 2018). The regulation of miRNAs into a network has gradually become an important mechanism for regulating gene expression (Fang et al., 2018). More and more studies have confirmed that miRNAs play an important role in the differentiation of neurons, such as the transmission of neurotransmitters, the occurrence of neuromorphology, and the formation of synaptic plasticity (Xu et al., 2017). Therefore, the value of miRNAs in depressive disorders is worth exploring. Numerous studies have shown that miR‐345‐5p is related to the development of many diseases, and its expression and role in different diseases are also different (Eilam‐Frenkel et al., 2018; Tinay et al.,). At present, the expression, role, and target of miR‐345‐5p in depression are still unclear. In recent years, several studies have found that miRNAs play a biological role by regulating downstream target genes (Lotfi et al., 2017). Cytokine inhibitory signaling protein (SOCS) is a specific receptor expressed on the cell surface, and activation or high expression of this receptor can significantly reduce the secretion of inflammatory factors by cells (Kedzierski et al., 2017). In microglia and macrophages, high expression of SOCS1 inhibits excessive activation of microglia (Zhang, Gao, et al., 2017). Therefore, it was speculated that miR‐345‐5p may regulate the progression of depression through SOCS1.

The main purpose of this study was to explore the mechanism of miR‐345‐5p in regulating of depression and provide a theoretical basis for finding new drug targets.

## 2 METHOD

### 2.1 Cell culture

The BV‐2 microglia cell line was got from the Kebai Culture Collection (Nanjing, China), and the HT22 hippocampal neuronal cell line was obtained from the Jining Culture Collection (Shanghai, China). All cells were cultured in RPMI 1640 medium containing 10% fetal bovine serum (FBS, Jitai).

### 2.2 Preparation of microglia conditioned medium

BV‐2 microglia were inoculated on serum‐free/glucose‐free DMEM for 1 hr in an anoxic environment. The BV‐2 microglia were then transferred to an anoxic incubator for 48 hr, and MCM was centrifuged. Microglia conditioned medium (MCM) was diluted to 1:1 with serum‐free medium.

### 2.3 Cell grouping and transfection

Four treatment groups were prepared in the present study, including the control group (BV‐2 microglial cells or hippocampal neuron cells), mimic control (NC) group (BV‐2 microglial cells or hippocampal neuron cells transfected with mimic control; Shanghai GenePharma Co., Ltd., Shanghai, China), miR‐345‐5p mimics group (BV‐2 microglial cells or hippocampal neuronal cells transfected with miR‐345‐5p mimics; Shanghai GenePharma Co., Ltd.), and lipopolysaccharide (LPS) group. BV‐2 microglial cells or hippocampal neuron cells were treated with LPS (10 μg/ml; dissolved in PBS; Beijing Solarbio Science & Technology Co. Ltd., Beijing, China) for 24 hr at 37°C. Cells were seeded into 6‐well plates at a density of 1 × 104 cells/well. The cells were starved overnight and then transfected with 75 pmol mimic control or miR‐345‐5p mimic using Lipofectamine^®^ 3000 (Invitrogen; Thermo Fisher Scientific, Inc.), according to the manufacturer's protocol. The cells were harvested 24 hr after transfection and then used for subsequent experiments.

Overexpression of suppressor of cytokine signaling 1 (SOCS1) was induced by transfecting cells (1 × 105 cells/ml) for 24 hr at 37°C with 2.5 μg pcDNA3.1‐SOCS1 plasmid (Shanghai GenePharma Co., Ltd, Shanghai, China) using Lipofectamine^®^ 3000 (Invitrogen; Thermo Fisher Scientific, Inc), according to the manufacturer's protocol. pcDNA3.1 served as the negative control.

### 2.4 Cell viability analysis

Cell proliferation was analyzed by Cell Counting Kit‐8 (CCK‐8; Dojindo). The absorbance values were finally determined at 450 nm using a microplate reader (SAFAS Xenius XL, Ruixuan).

### 2.5 ELISA

ELISA kit was used to detect the levels of IL‐10, IL‐6, TNF‐β, TNF‐α, and indoleamine 2,3‐dioxygenase 1 (IDO1) (Elabscience, Wuhan, China). The culture supernatant was added to a 96‐well plate. 100 μl of biotinylated antibody was added. The chromogenic substrate was incubated for 15 min. The stop solution was incubated for 10 min. Optical density (OD) values were used to detect at 450 nm using a microplate reader (SAFAS Xenius XL, Ruixuan).

### 2.6 Apoptosis detection

In apoptosis analysis, transfected cells were harvested and stained with Annexin V‐FITC‐PI Assay Kit (Solarbio). Analysis was performed using a FACS can flowcytometer (BD Biosciences, Keyuxingye).

### 2.7 Cell cycle detection

The cells were subjected to conventional subculture for about 18 hr for corresponding experimental treatment, and then, the cells were blown into a single cell suspension. Cells were collected by centrifugation and then resuspended with stationary liquid (70% ethanol, 0.1% Triton X‐100, 2 g/ml RNase A). They were fixed at room temperature for 2 hr and centrifuged at 1,000 × *g* and 4°C for 5 min. After that, cells were resuspended in 500 μl PBS containing 10 g/ml PI. The cell cycle was analyzed by flow cytometry (BD Accuri C6 cytometer; BD Biosciences, ANNORON, Beijing, China).

### 2.8 Luciferase reporter gene assay

Wild‐type SOCS 1‐3ʹ‐UTR (WT) and mutant SOCS 1‐3ʹ‐UTR (MT) containing the putative binding site of miR‐345‐5p were constructed. A reporter vector containing WT or MT SOCS1 3ʹ‐UTR was cotransfected into BV‐2 cells with miR‐345‐5p mimic or NC using Lipofectamine 2000. After transfection 48 hr, the activity of luciferase was analyzed by double luciferase analysis system (Promega).

### 2.9 Western blot analysis

The transfected cells were collected, total protein was extracted, and protein concentration was quantified using the BCA Protein Assay Kit. Then, it was incubated with rabbit anti‐TNF‐α (1:1,000; GenePharma), TNF‐β (1:1,000; GenePharma), IDO1 (1:500; GenePharma), apoptosis regulator Bax (1:1,000; GenePharma), apoptosis regulator Bcl2 (1:1,000; GenePharma), PR‐caspase‐3 (1:1,000; GenePharma), CuraveDcAsAsE3 (1:1,000; GenePharma), SOCS1 (1:500; GenePharma), and GAPDH (1:5,000; GenePharma) overnight at 4°C. After that, 1:5,000 labeled anti‐rabbit secondary antibody was added for 1 hr. The specific experimental methods for Western blot analysis were performed with reference to the literature (Swets et al., 2018).

### 2.10 Reverse transcription–quantitative polymerase chain reaction (QRT‐PCR) analysis

Total RNA in cells was extracted using TRIzol reagent (Huamai, Beijing, China). qRT‐PCR was performed using a ViiA^TM^ 7 real‐time PCR system (Jinuo, Shanghai, China). GAPDH and U6 were used as internal references. The expression levels of miR‐345‐5p were calculated by the 2‐DDCt method. qRT‐PCR specific experimental methods were performed with reference to the literature (Zhang et al., 2018). Primer sequences were shown in Table S1.

### 2.11 Statistical methods

The monitoring data were analyzed by SPSS19.0 statistical software. The results of data analysis were shown as mean ± standard deviation (mean ± *SD*). Multigroup data analysis was based on one‐way ANOVA. LSD test was used for subsequent analysis. *p* < .05 indicated the difference was significant.

### 2.12 Ethical statement

Not applicable.

## 3 RESULT

### 3.1 miR‐345‐5p inhibits the cell proliferation of BV‐2 microglial cells

**FIGURE 1:**
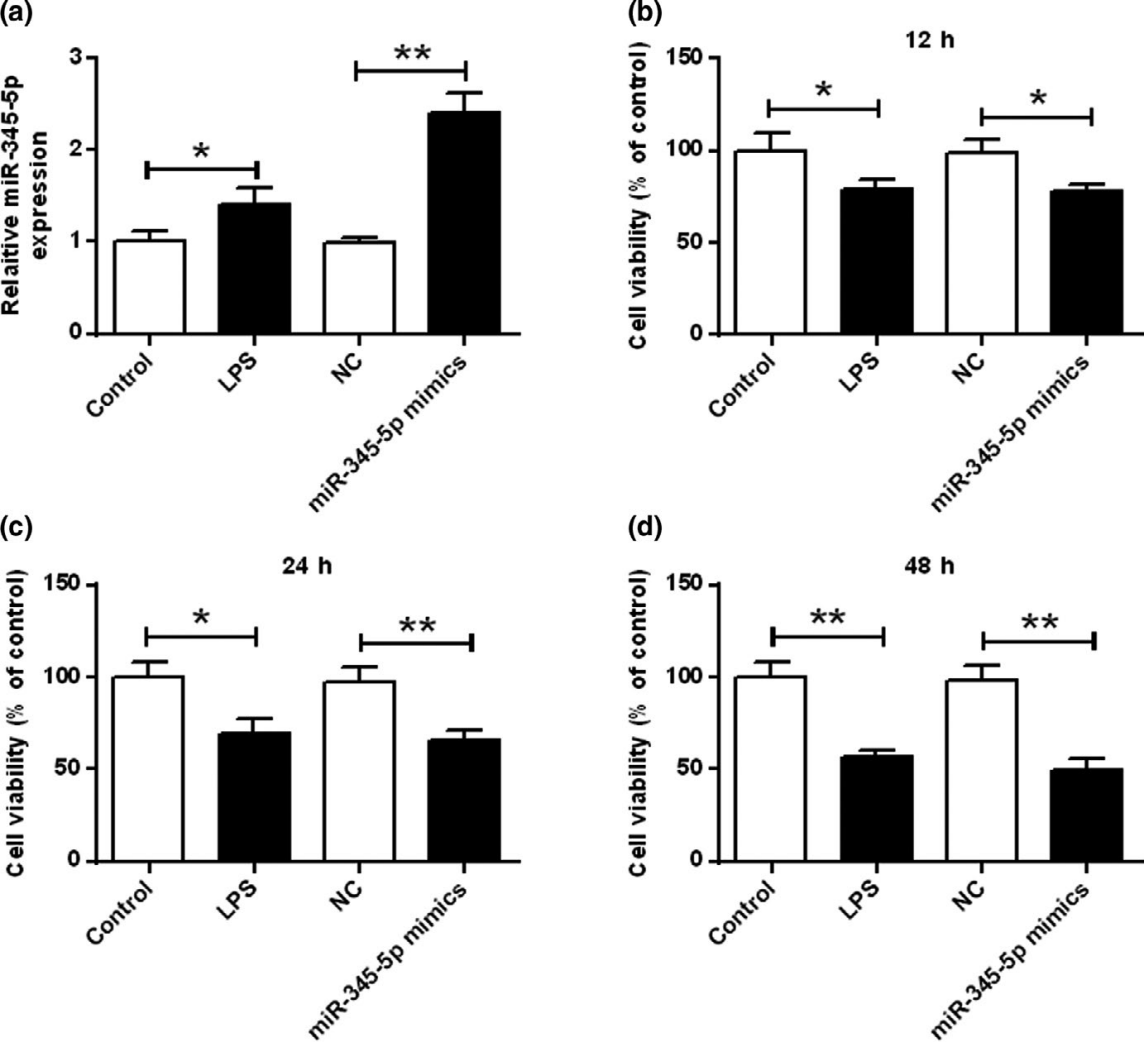
Effect of miR‐345‐5p on cell viability of BV‐2 microglia. (a) mRNA expression level of miR‐345‐5p in BV‐2 microglia. (b–d) Effect of miR‐345‐5p on cell viability of BV‐2 microglia at different times.*p < .05, **p < .01

As shown in Figure 1a, contrasted with the control group, the expression level of miR‐345‐5p was upregulated after LPS stimulation (*p* < .05). After miR‐345‐5p mimics were transfected into BV‐2 microglia, contrasted with the NC group, the expression level of miR‐345‐5p was significantly increased in the miR‐345‐5p mimic group (*p* < .01). In addition, as shown in Figure 1b–d, contrasted with control group, the viability of BV‐2 microglia was decreased with the prolongation of treatment time after LPS stimulation. Contrasted with NC group, the mimic of microRNA‐345‐5p could significantly reduce the viability of BV‐2 microglia (*p* < .05, *p* < .01). The results indicated that miR‐345‐5p can reduce the cell viability of BV‐2 microglia.

### 3.2 miR‐345‐5p raised the expression levels of pro‐inflammatory cytokines in BV‐2 microglial cells

**FIGURE 2:**
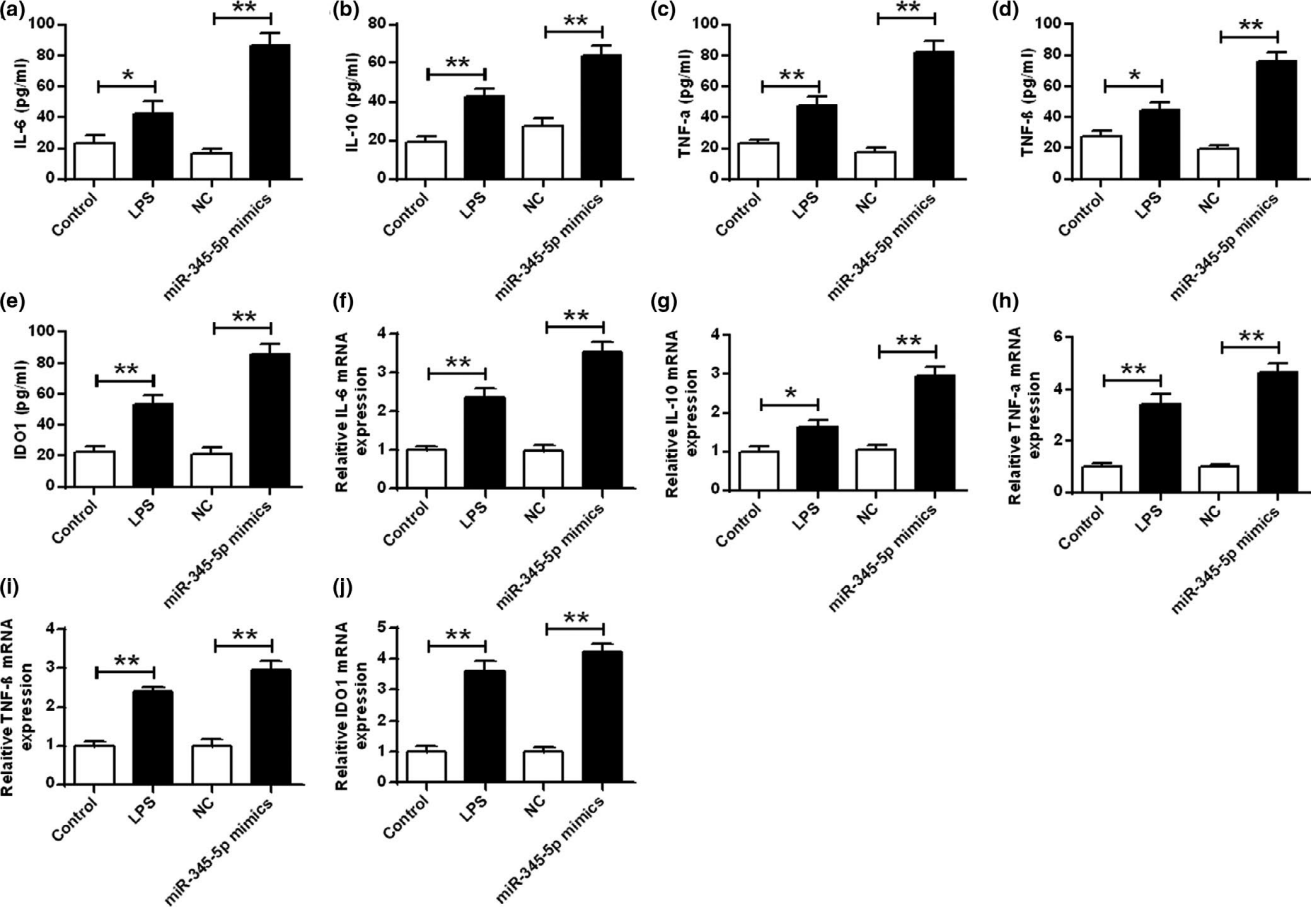
Effect of miR‐345‐5p on the expression levels of pro‐inflammatory cytokines in BV‐2 microglia. (a–e) ELISA was used to measure the expression levels of IL‐6, IL‐10, TNF‐α, TNF‐β, and IDO1 in BV‐2 microglia. (f–j) qRT‐PCR was used to measure the expression levels of IL‐6, IL‐10, TNF‐α, TNF‐β, and IDO1 in BV‐2 microglia. *p < .05, **p < .01

Next, the relationship between miR‐345‐5p was analyzed. As shown in Figure 2, IL‐6, TNF‐α, TNF‐β, IL‐10 and IDO1 levels and mRNA levels were increased after LPS stimulation contrasted with that in the control group (*p* < .05). Contrasted with the control group, mRNA levels of IL‐6, TNF‐β, TNF‐α, IL‐10, and IDO1 transfected with miR‐345‐5p mimics were significantly increased (*p* < .01).

In addition, the protein levels of TNF‐β, TNF‐α, and IDO1 were increased after LPS stimulation contrasted with that in the control group (*p* < .05). Contrasted with the NC group, TNF‐β, TNF‐α, and IDO1protein levels were significantly increased in the BV‐2 microglia transfected with miR‐345‐5p mimics (*p* < .01; Figure S1).

### 3.3 miR‐345‐5p contributed to microglial‐induced injury and apoptosis of hippocampal neuron cells

**FIGURE 3:**
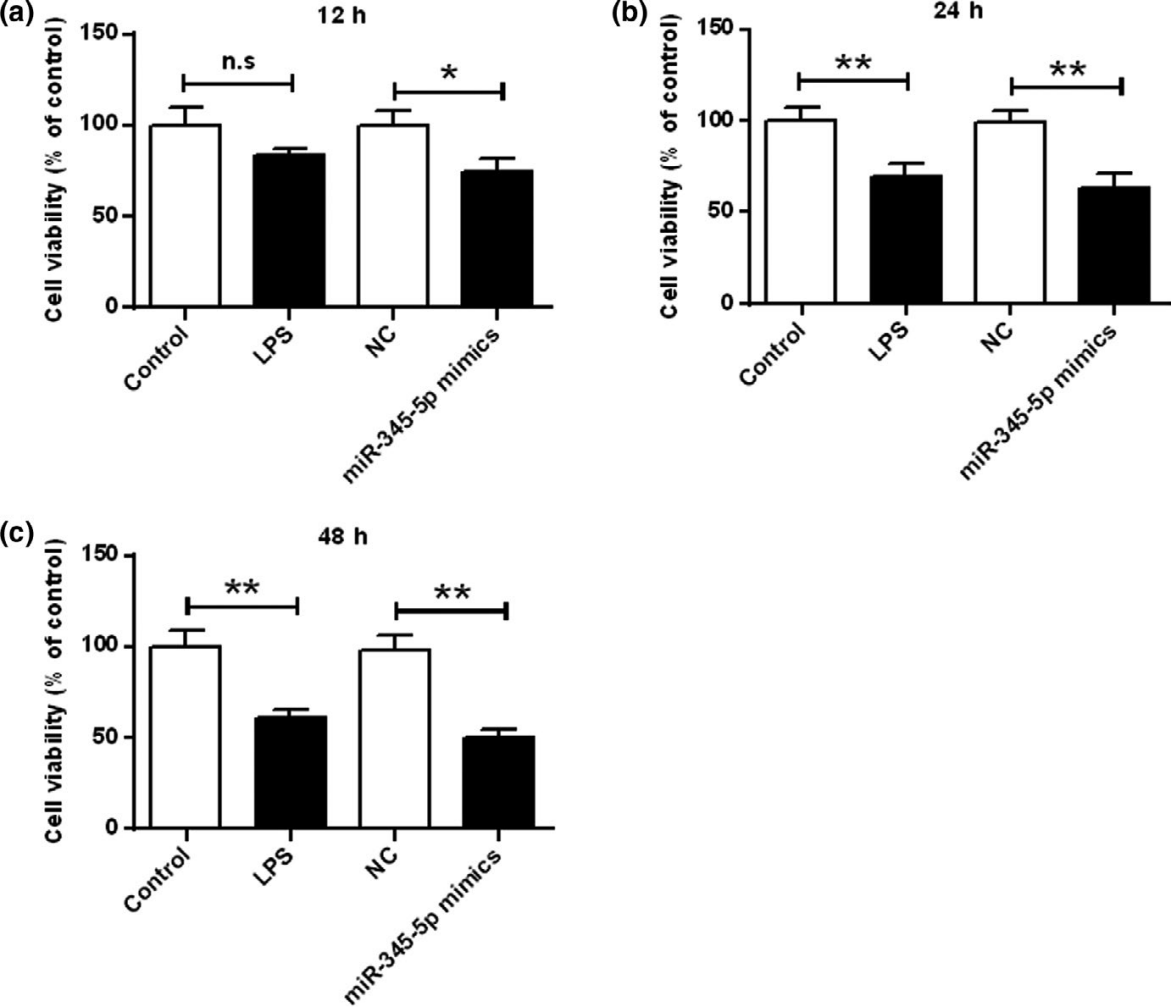
Effect of miR‐345‐5p on cell viability of hippocampal cells at different times. *p* < .05, **p < .01

As shown in Figure 3, contrasted with the control group, the LPS stimulation reduced the cell viability of hippocampal neurons. The cell viability of hippocampal neurons was significantly reduced in the miR‐345‐5p mimic group (*p* < .05, *p* < .01). The results demonstrated that miR‐345‐5p reduced the cell viability of hippocampal neurons induced by microglia.

### 3.4 miR‐345‐5p contributed to apoptosis of hippocampal neuron cells

As shown in Figure S2, contrasted with the control group, LPS stimulation induced apoptosis of hippocampal neurons. Contrasted with NC group while, miR‐345‐5p mimic significantly induced apoptosis of hippocampal neurons (*p* < .01). These data demonstrated that miR‐345‐5p stimulated microglia to induce apoptosis in hippocampal neurons.

**FIGURE 4:**
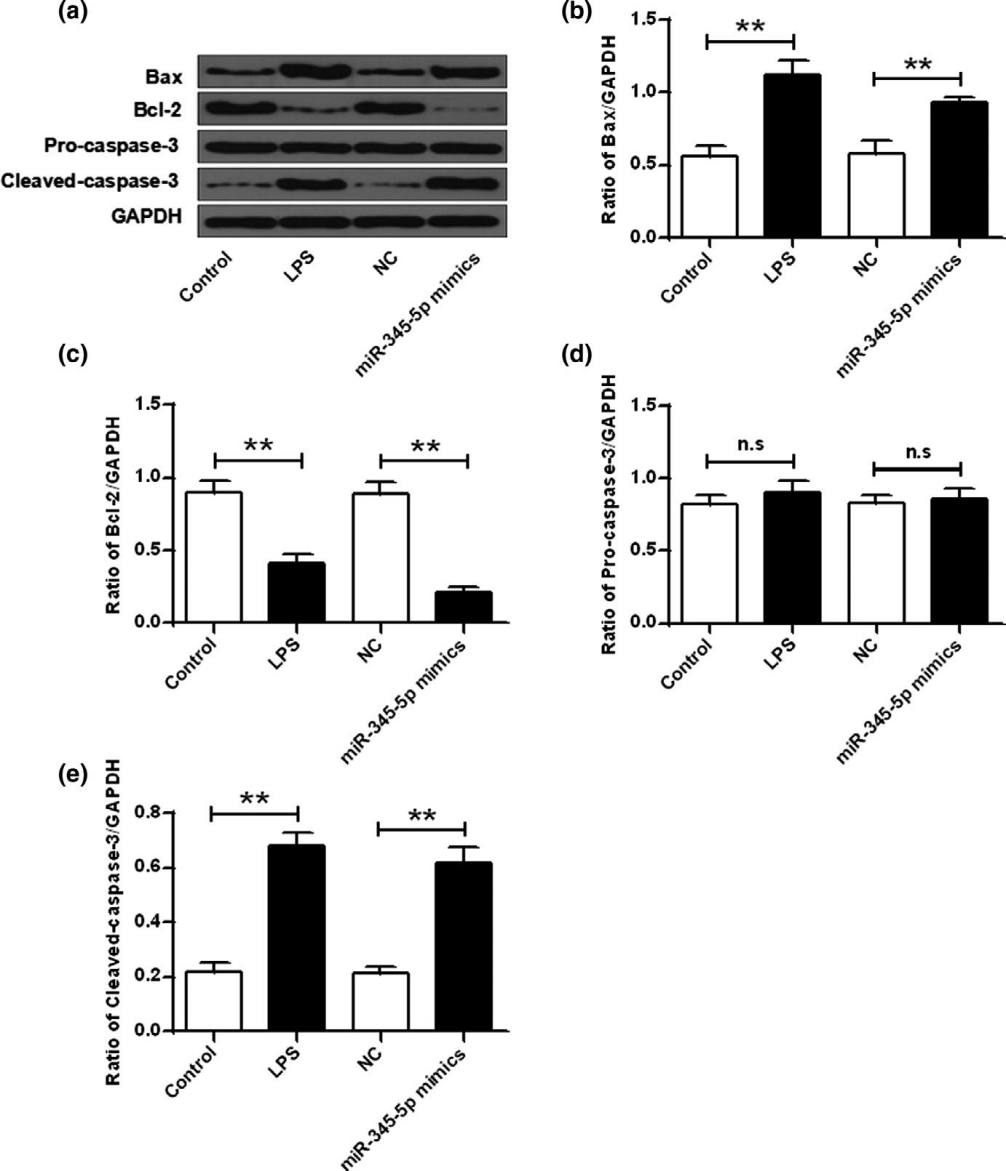
Effect of miR‐345‐5p on the expression of apoptosis‐related proteins. (a) Protein expression levels of Bax, Bcl‐2, pre‐caspase‐3, and cleaved caspase‐3. (b–e) Quantitative densitometry of expression levels of Bax, Bcl‐2, pro‐caspase‐3, and cleaved caspase‐3. Ns, no meaning. **p < .01

As shown in Figure 4, the expression of Bcl‐2 in hippocampal neurons was significantly decreased after LPS stimulation, Bax, and cleaved caspase‐3 were significantly raised, and the expression levels of pro‐caspase‐3 were not significantly different contrasted with that in the control group. Contrasted with the NC group, the expression level of Bcl‐2 in hippocampal neurons was significantly decreased, the expression levels of Bax and cleaved caspase‐3 were significantly increased, and the expression level of precaspase‐3 was not significantly different. It was shown that miR‐345‐5p induced hippocampal neuronal apoptosis by Bax, Bcl‐2, and cleaved caspase‐3.

### 3.5 miR‐345‐5p promotes the cell cycle arrest of hippocampal neuron cells

The results showed that contrasted with the control group, LPS‐treated microglia MCM reduced the number of cells in the G1 phase and increased the number of cells in the S phase (*p* < .05). Contrasted with the NC group, the miR‐345‐5p mimic significantly reduced the number of cells in the G1 phase and significantly raised the number of cells in the S phase (*p* < .01; Figure S3). These results demonstrated that miR‐345‐5p can trigger S‐phase arrest in hippocampal neuronal cells.

### 3.6 miR‐345‐5p targets directly with the 3′‐UTR region of SOCS1

**FIGURE 5:**
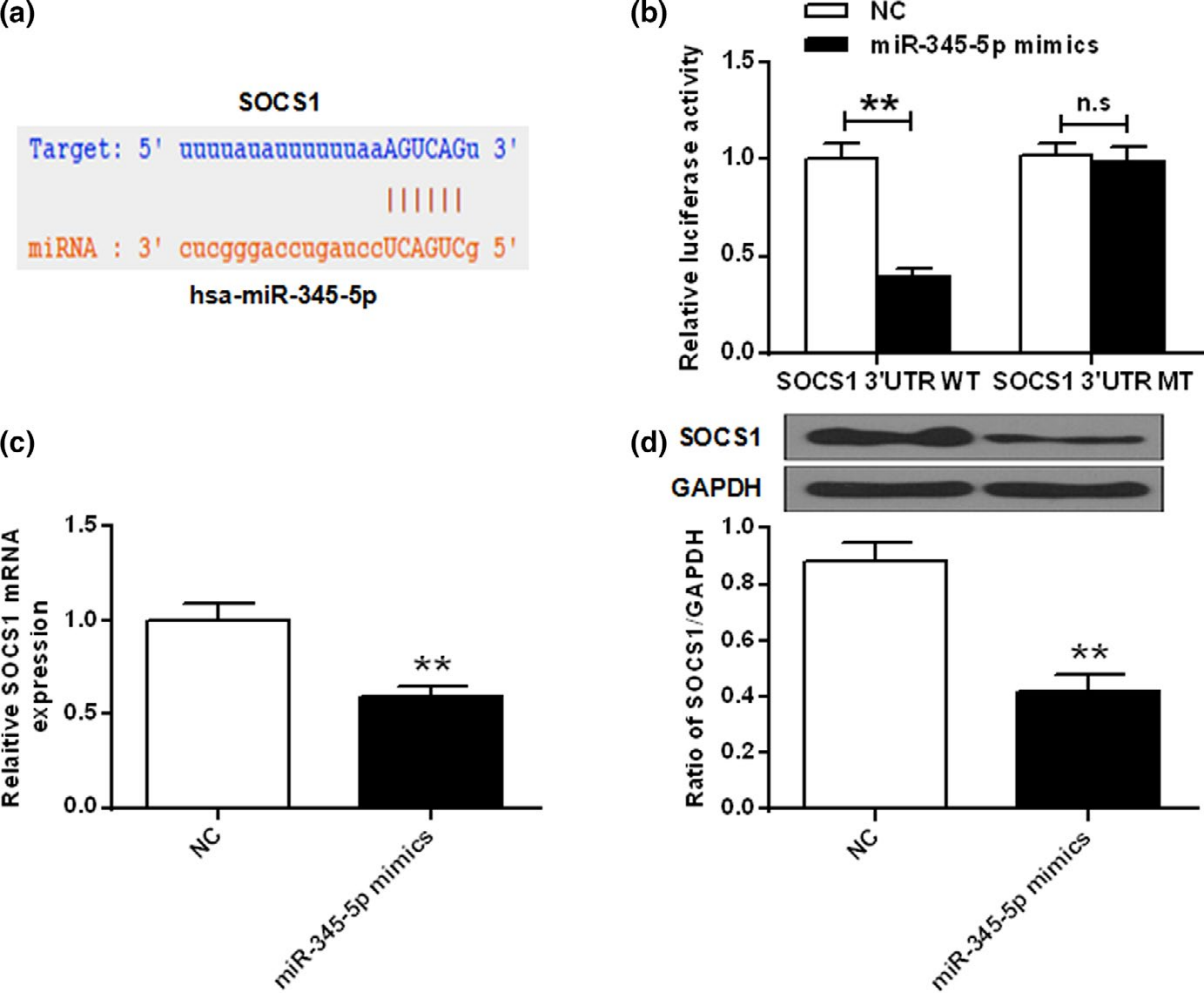
MiR‐345‐5p directly targeted the 3ʹ‐UTR region of SOCS1. (a) Binding site of miR‐345‐5p and SOCS1. (b) Luciferase activity was detected by a luciferase reporter assay. (c) mRNA expression level of SOCS1 in HEK 293 cells after miR‐345‐5p mimic treatment. (d) SOCS1 protein levels in miR‐345‐5p mimetic treated HEK 293 cells. Ns, no meaning. **p < .01

The underlying mechanisms by which miR‐345‐5p regulates depression were explored. We predicted by online prediction tool starBase v2.0 and SOCS1 was identified as a potential target for miR‐345‐5p (Figure 5a). The luciferase reporter gene assay showed that the luciferase activity of pGL3‐REPOR‐miR‐345‐5p‐WT was reduced by the SOCS1 mimetic, but the luciferase activity of pGL3‐REPOR‐miR‐345‐5p‐mut had not significant changes (Figure 5b). In addition, as shown in Figure 5c,d, the mRNA and protein expression levels of SOCS1 in the miR‐345‐5p mimics group were significantly reduced contrasted with that in the NC group. These results indicated that miR‐345‐5p may exert its biological function through SOCS1.

### 3.7 SOCS1 plays a part in the effect of miR‐345‐5p

**FIGURE 6:**
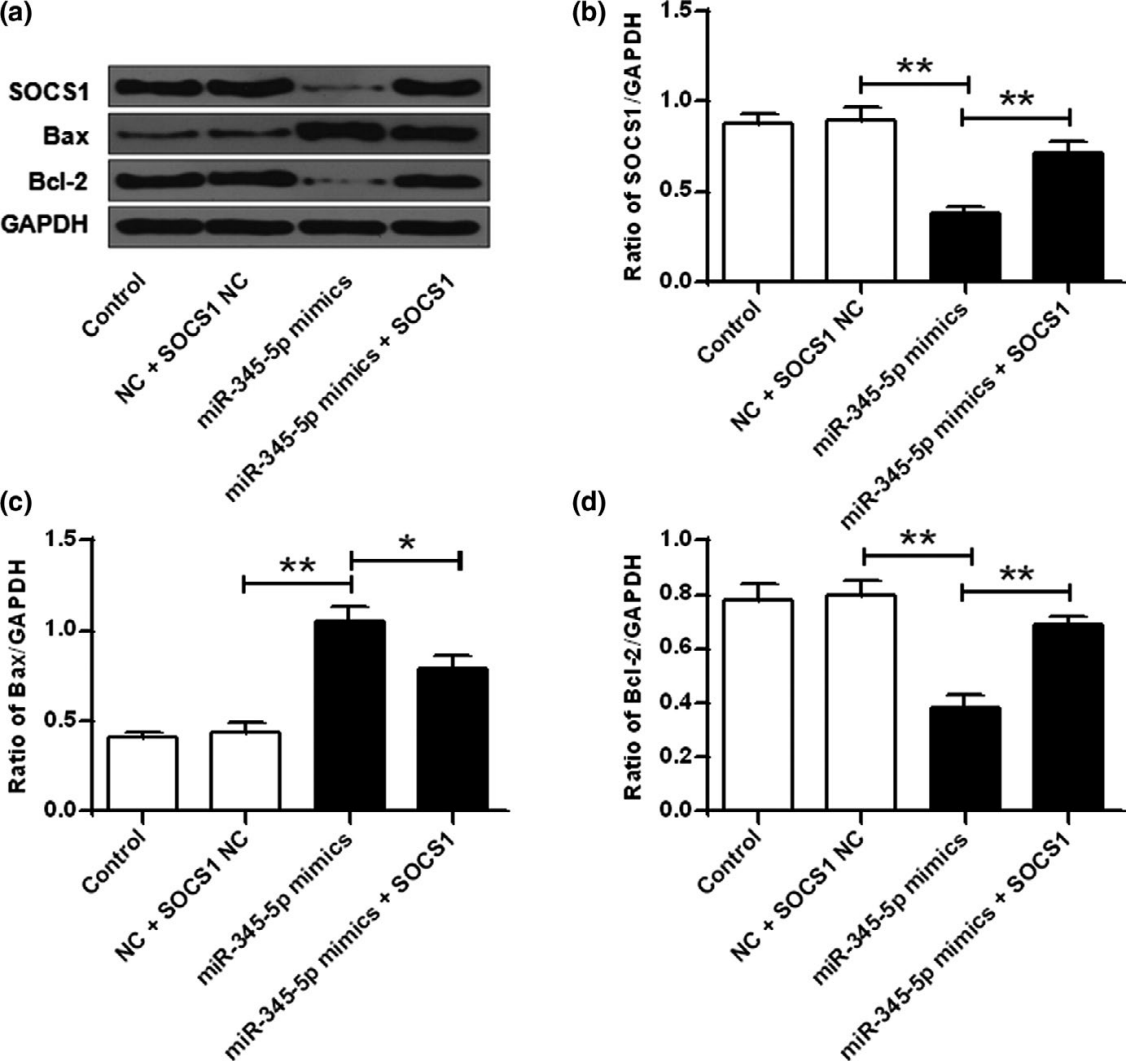
miR‐345‐5p exerted a biological role through SOCS1. (a) Protein expression levels of SOCS1, Bax, and Bcl‐2. (b) Quantitative densitometry of SOCS1 expression levels. (c) Quantitative densitometry of Bax expression levels. (d) Quantitative densitometry of Bcl‐2 expression levels. *p < .05, **p < .01

To further analyze whether miR‐345‐5p exerts a biological effect on hippocampal neurons through SOCS1, miR‐345‐5p mimics and SOCS1 were cotransfected into hippocampal neurons. The results showed that the miR‐345‐5p mimic significantly reduced the protein expression levels of SOCS1 and Bcl‐2 contrasted with that in the control group and the NC group, and significantly raised the protein expression level of Bax (*p* < .01). In addition, cotransfection of miR‐345‐5p mimic with SOCS1 reversed the effect of miR‐345‐5p mimics on SOCS1, Bax, and Bcl‐2 (*p* < .05, *p* < .01; Figure 6). These results demonstrated that miR‐345‐5p mediated hippocampal neuronal cell damage via SOCS1.

## 4 DISCUSSION

Depression is a comprehensive feature characterized by significant and lasting emotional disorders (Ogundele, 2018). It has the characteristics of high morbidity, disability, and suicide rate (Wang et al., 2018). In 2020, the number of people with functional disabilities caused by depression will rise to the second place in all diseases, next only to cardiovascular diseases, and will account for one third of the global causes of disabilities caused by neuropsychiatric factors. By 2030, depression will become the disease with the highest incidence (Krogh, Nordentoft, & Sterne, 2011). It will become the most important mental disorder to be solved. However, there is currently limited understanding of the molecular mechanisms of its pathogenesis, and there is still a lack of effective and low‐potency therapeutic drugs. Recently, a large number of studies have also found that cytokines secreted by microglia in the brain are also associated with mood disorders, and the development of new antidepressants as a target is becoming a research hotspot in this field (Wiener et al., 2017).

In this study, we aimed to investigate the roles and mechanisms of miR‐345‐5p in TRD, with a focus on BV‐2 microglial cells and HT22 hippocampal neuron cells. Activated microglia possess in CNS diseases (such as depression) can perform phagocytosis and release specific cytokines, including pro‐inflammatory cytokines and neurotrophic factors, resulting in inflammatory reactions in the nervous system, exacerbate damage to nerve cells (Wang, Zhang, et al., 2015). And the hippocampus has been considered as a core factor in the pathophysiology of depression. Also, the microglia can mediate nerve damage in CNS diseases (Campbell & Macqueen, 2004). On the basis of the pivotal role of microglia and hippocampus in CNS‐related diseases, these two cells were selected as the research models in the present study.

miRNAs are widely distributed in various tissues and organs, and participate in the regulation of various life activities including development, immune response, hematopoiesis, and metabolism, and are important for maintaining the normal physiological functions of the body. Analysis of miRNA microarrays reveals abnormal expression of miRNAs in the prefrontal cortex of depressed suicide populations (Lopez et al., 2014). Analysis of downstream targets of differentially expressed miRNAs reveals that a large proportion of them were associated with depression, and antidepressant treatment reversed the expression of miRNAs (Pandey, Rizavi, Zhang, Bhaumik, & Ren, 2018). In addition, some studies have found that miR‐144‐5p was significantly lower in patients with depression than in normal subjects, and its expression level is associated with depressive symptoms (Wang, Sundquist, et al., 2015). It can be seen that miRNA is closely related to depression and participates in the process of hippocampal neuron production. As a member of miRNA, miR‐345‐5p differs in its role in different diseases (Eilam‐Frenkel et al., 2018). In this study, it was found that miR‐345‐5p was significantly increased in BV‐2 microglia (*p* < .01; Figure 1a). In addition, miR‐345‐5p mimics were able to reduce the cell viability of BV‐2 microglia. The results indicated that miR‐345‐5p can reduce the cell proliferation of BV‐2 microglia. Moreover, miR‐345‐5p stimulates microglia to decrease the activity of hippocampal neurons, induce apoptosis, and induce cell cycle arrest in S phase, so it can control the development of depression by inhibiting the expression of miR‐345‐5p.

In recent years, foreign psychology studies on depression have shown that depression is associated in the immune system (Mcguinness & Harkin, 2015). More recent studies suggest that depression is accompanied by immune activation, which is characterized by excessive secretion of cytokines, accompanied by acute phase reactions, increased peripheral blood mononuclear cells and neutrophil counts, and increased autoantibody titers. IL‐10, TNF‐α, IDO1, and IL‐6 and TNF‐β are typical cellular inflammatory factors (Arad, Piontkewitz, Albelda, Shaashua, & Weiner, 2017; Farup, Hestad, Lydersen, Rudi, & Ueland, 2017). Activated microglia possess in CNS diseases (such as depression) can perform phagocytosis and release specific cytokines, including pro‐inflammatory cytokines and neurotrophic factors, resulting in inflammatory reactions in the nervous system, exacerbate damage to nerve cells (Properzi et al., 2015). And the hippocampus has been considered as a core factor in the pathophysiology of depression (Zhang, Yuan, Pu, Yang, & Xie, 2017). Also, the microglia can mediate nerve damage in CNS diseases (Guo, Chang, Li, Li, & Wang, 2016). On the basis of the pivotal role of microglia and hippocampus in CNS‐related diseases, these two cells were selected as the research models in the present study. The results here demonstrated that miR‐345‐5p increased the cell viability of BV‐2 microglia and the expression level of pro‐inflammatory cytokines. The conditioned medium of microglia treated with miR‐345‐5p reduced the cell viability and apoptosis of HT22 hippocampal cells. Collectively, the present research demonstrated that miR‐345‐5p mediated inflammatory injury in hippocampal neuron cells via the activation of microglial cells.

In recent years, there have been reports of miRNA targeting and regulation related pathways, which affect the occurrence and development of diseases (Niu et al., 2017). SOCS1 is a cytokine signaling inhibitor that plays a part in the regulation of a variety of cytokines, growth factors, and hormones (Qian, Lv, & Li, 2018). SOCS1 contains a highly conserved SH2 domain that recognizes different molecules, competitively binds to phosphorylated tyrosine at the receptor, inhibits signal transduction activation, and participates in a variety of signal transduction pathways (Yu, Peng, Schlee, Barchet, Eishübinger, et al., 2018). Cytokine (CK) signaling pathway and Toll‐like receptor (TLR) signaling pathway play an important role in regulating the proliferation, differentiation, and survival of immune cells, as well as maintaining immune homeostasis (Wahid, Rafique, Saleem, Ali, & Idrees, 2018). In cells, the above signaling pathway is strictly regulated by a number of negative regulatory molecules, among which the most important one is cytokine signaling inhibitory protein 1 (SOCS1), a JAK‐binding protein, and it can inhibit CK and TLR signal pathway by multitarget intervention, so that the signal cascade can be terminated in time (Yu, Peng, Schlee, Barchet, & Novak, 2018). The role of it in depression is not clear. In this study, it was found that SOCS1 was a potential target for miR‐345‐5p. The miR‐345‐5p mimetic significantly reduced the expression levels of SOCS1 and Bcl‐2, and significantly upregulated the expression level of Bax. Cotransformed of miR‐345‐5p mimetic with SOCS1 reversed the effect of miR‐345‐5p mimic on the expression levels of Bax, SOCS1, and Bcl‐2 proteins. These results demonstrated that miR‐345‐5p was able to regulate hippocampal neuronal cell damage via SOCS1. In future research, we would try to assess the related signal pathways regulated by miR‐345‐5p/SOCS1 in further studies to expand and facilitate the understanding of the roles of miR‐345‐5p in depression.

## 5 CONCLUSION

MiR‐345‐5p induced inflammatory damage in hippocampal neurons by activating microglia. It may be an effective target for TRD therapy. It would provide experimental basis for the clinical prognosis of the disease and further targeted intervention treatment research.

## CONFLICT OF INTERESTS

The authors declare that they have no competing interests.

## AUTHOR CONTRIBUTION

All the authors have contributed to the study design, experiments, data analysis, and manuscript preparation.

## CONSENT FOR PUBLICATION

All authors have read and approved the final manuscript.

## Supporting information

Figure S1

Figure S2

Figure S3

Table S1

## Data Availability

The analyzed data sets generated during the study are available from the corresponding author on reasonable request.
