# CORRIGENDUM

**DOI:** 10.1002/brb3.2149

**Published:** 2021-06-16

The article by Liu et al. (2020) was published in BRB3:10:9 issue with the below error

The author affiliation is shown as:

“Yulan Liu, Jun Yu, Xinrui Wang, Jicheng Dong”

Psychiatric Department, Qingdao Mental Health Center, Qingdao University, Qingdao City, China

This should have appeared as:

“Yulan Liu^1^, Jun Yu^2^, Xinrui Wang^1^, Jicheng Dong^1^”

^1^Psychiatric Department, Qingdao Mental Health Center, Qingdao University, Qingdao City, China

^2^Psychiatric Department, Laizhou Glory Solider's Hospital in Yantai City, Laizhou, 261400, China
