# Supplementary figures and images for: MicroRNA‐345‐5p regulates depression by targeting suppressor of cytokine signaling 1
Source: Brain Behav. 2020 Jul 30;10(9):e01653. doi: 10.1002/brb3.1653 (PMC7507044; doi:10.1002/brb3.1653)

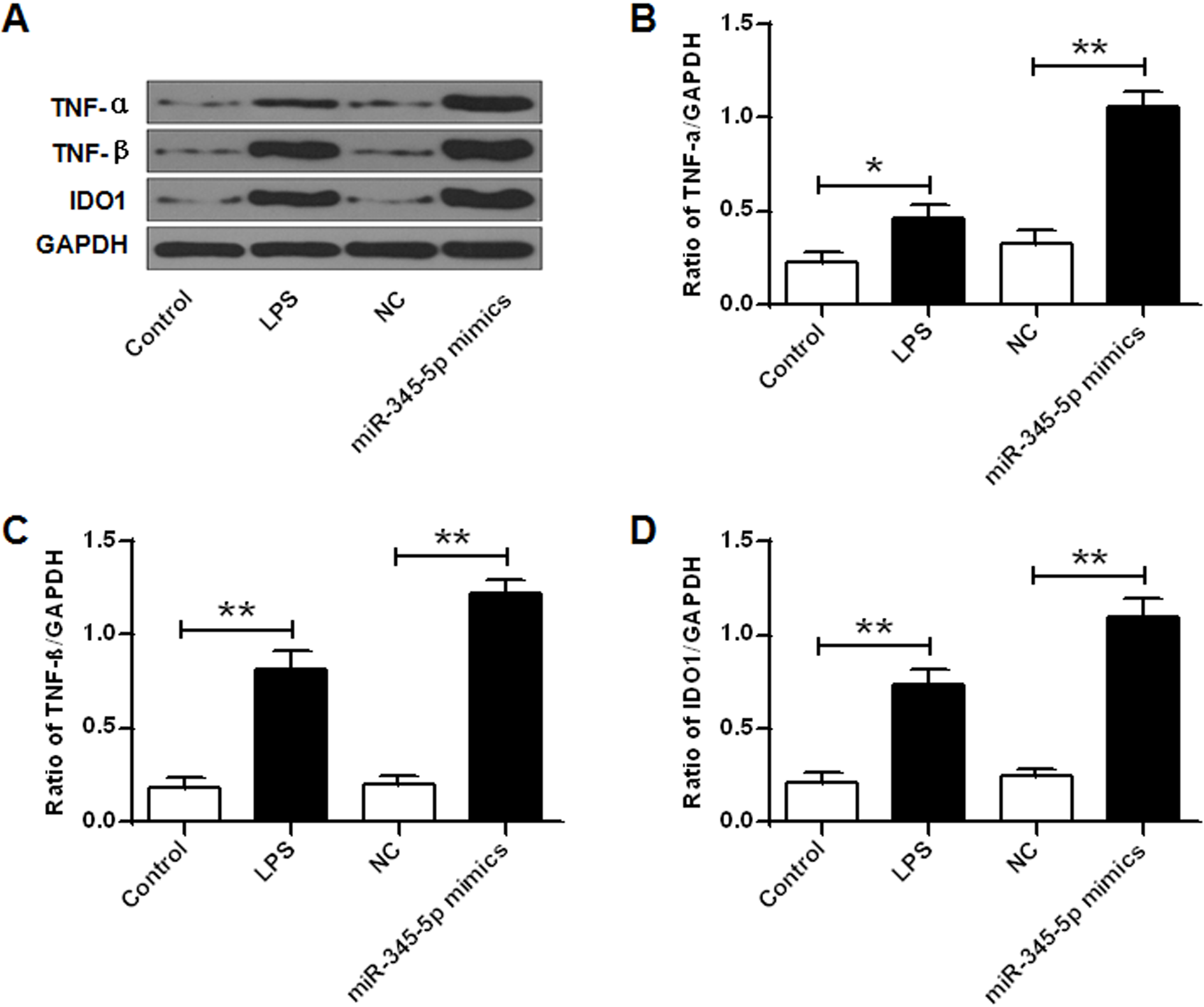

Supplement: Supplementary file 1 — Figure S1 [file BRB3-10-e01653-s001.tif]

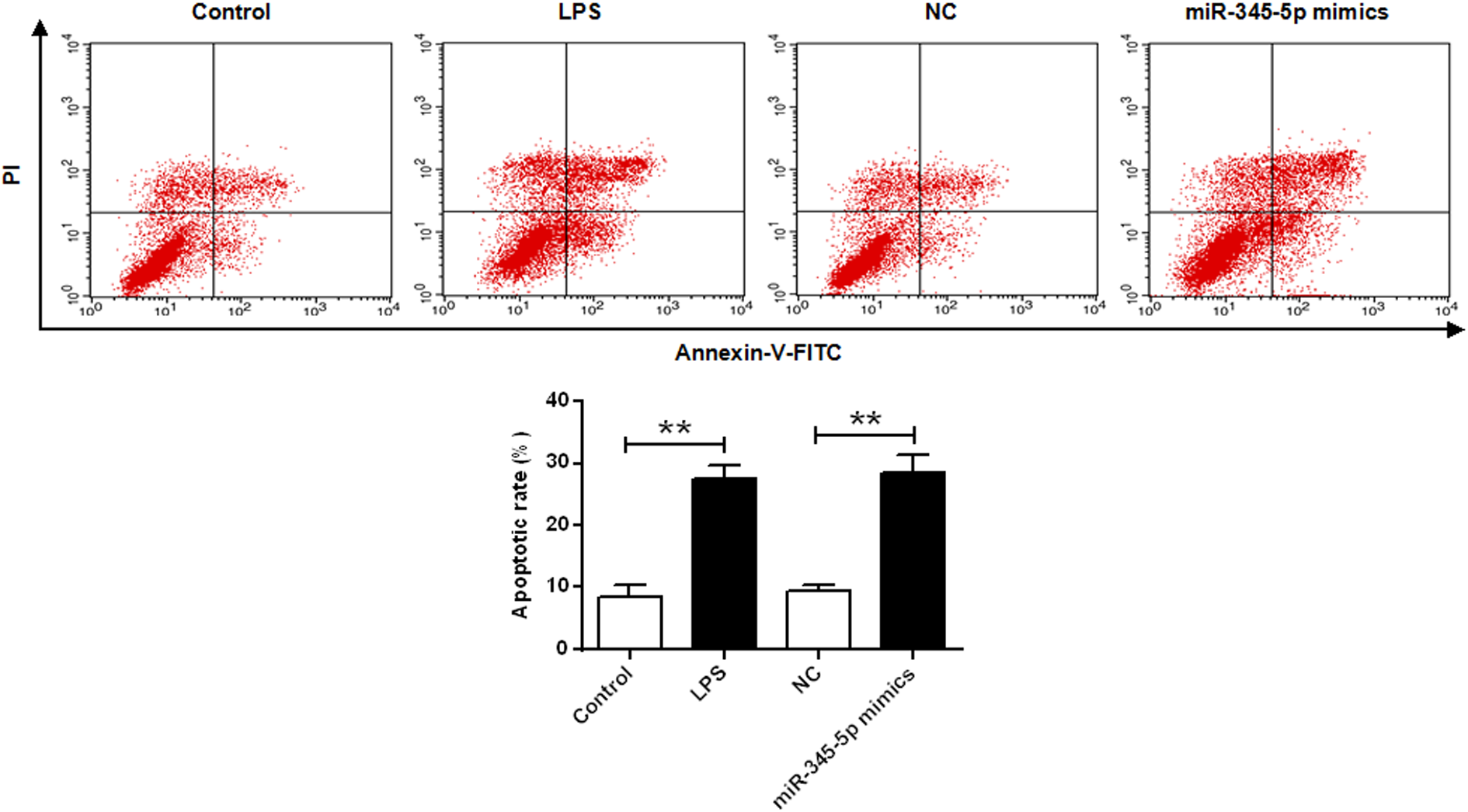

Supplement: Supplementary file 2 — Figure S2 [file BRB3-10-e01653-s002.tif]

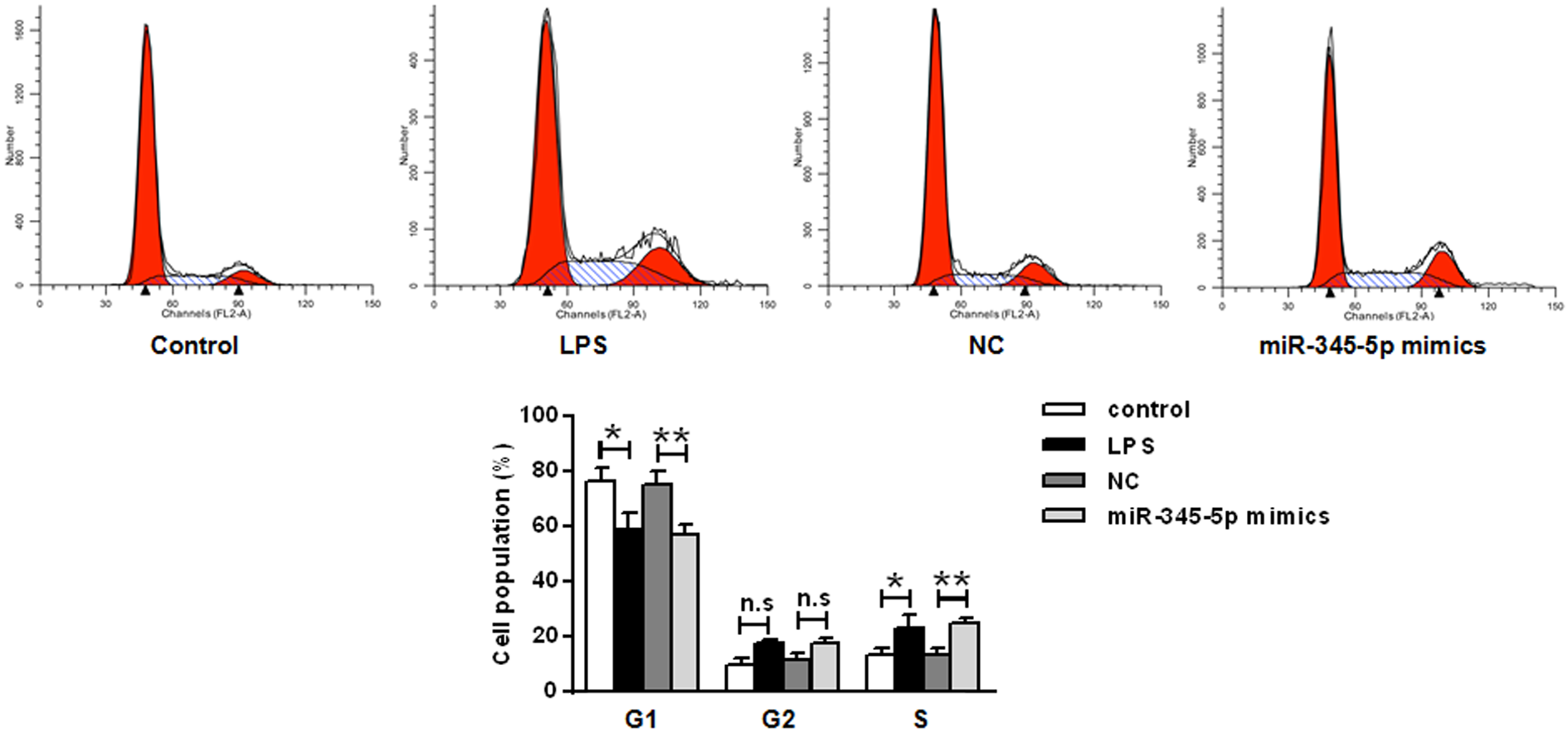

Supplement: Supplementary file 3 — Figure S3 [file BRB3-10-e01653-s003.tif]
