# Supplementary material for: MicroRNA‐345‐5p regulates depression by targeting suppressor of cytokine signaling 1
Source: Brain Behav. 2020 Jul 30;10(9):e01653. doi: 10.1002/brb3.1653 (PMC7507044; doi:10.1002/brb3.1653)
Supplement: Supplementary file 4 — Table S1 [file BRB3-10-e01653-s004.docx]

**Table S1**

Sequences of primers used in qRT-PCR

| Gene | Forward primer (5’-3’) | Reversed primer (5’-3’) |
| --- | --- | --- |
| miR-345-5p | GTCGTATCCAGTGCAGGGTCCGAGG | TATGCTGCTCGGGACCTGATCCTCA |
| U6 | GCAGGGTCCGAGGTATTCGCACTGG | ACAGTAGGAACGCGTCCCCGG |
| IL-6 | TTCTTGGGACTGATGCTGGT | CAAGTGCATCATCGTTGTTCA |
| IL-10 | AGTACAGCCGGGAAGACAAT | TTTCTGGGCCATGCTTCTCT |
| TNF-α | CAGAAAGCATGATCCGCGAC | GGTCTGGGCCATAGAACTGA |
| TNF-β | CATCCTGAAACCTGCTGCTC | GGAGGAAAAGAGCTGGACCT |
| IDO1 | GACTGTGTCCTGGCAAACTG | GTAGCTATGTCGTGCAGTGC |
| GAPDH | CGCTCTCTGCTCCTCCTGTT | CCATGGTGTCTGAGCGATGT |
